# Integrative Analyses of Genes Associated With Osteoporosis in CD16+ Monocyte

**DOI:** 10.3389/fendo.2020.581878

**Published:** 2021-01-21

**Authors:** Bin Hu, Xiangan Kong, Li Li, Fang Dai, Qiu Zhang, Ruifeng Shi

**Affiliations:** ^1^Department of Orthopedics, The Second People’s Hospital of Hefei, Hefei, China; ^2^Department of Endocrinology, The First Affiliated Hospital of Anhui Medical University, Hefei, China

**Keywords:** osteoporosis, peak bone mass, monocyte subsets, differentially expressed genes, Microarrary gene expression data

## Abstract

**Background:**

Osteoporosis is a metabolic bone disease characterized by decreased bone mineral density and abnormal bone quality. Monocytes can secret cytokines for bone resorption, resulting in bone mass loss. However, the mechanism by which monocytes subpopulations lead to osteoporosis remains unclear. The aim of this study was to identify genes associated with osteoporosis in monocytes subsets.

**Methods:**

Three microarray datasets including GSE7158 (transcription of low/high-peak bone mass), GSE101489 (transcription of CD16+/CD16− monocyte) and GSE93883 (miRNA expression profile of primary osteoporosis) were derived from the Gene Expression Omnibus (GEO) database and analyzed with GEO2R tool to identify differentially expressed genes (DEGs). Functional enrichment was analyzed using Metascape database and GSEA software. STRING was utilized for the Protein–Protein Interaction Network construct. The hub genes were screened out using the Cytoscape software. Related miRNAs were predicted in miRWalk, miRDB, and TargetScan databases.

**Results:**

Total 368 DEGs from GSE7158 were screened out, which were mostly enriched in signaling, positive regulation of biological process and immune system process. The hub genes were clustered into two modules by PPI network analysis. We identified 15 overlapping DGEs between GSE101489 and GSE7158 microarray datasets. Moreover, all of them were up-regulated genes in both datasets. Then, nine key genes were screened out from above 15 overlapping DEGs using Cytoscape software. It is a remarkable fact that the nine genes were all in one hub gene module of GSE7158. Additionally, 183 target miRNAs were predicted according to the above nine DEGs. After cross-verification with miRNA express profile dataset for osteoporosis (GSE93883), 12 DEmiRNAs were selected. Finally, a miRNA-mRNA network was constructed with the nine key genes and 12 miRNAs, which were involved in osteoporosis.

**Conclusion:**

Our analysis results constructed a gene expression framework in monocyte subsets for osteoporosis. This approach could provide a novel insight into osteoporosis.

## Introduction

Osteoporosis is a metabolic bone disease that causes bone mass loss and fragility fracture (1). It is known that postmenopausal women and elderly people are at higher risk for osteopenia and osteoporosis. Osteoporotic fractures, leading to serious physical, emotional, and financial consequences, are becoming an ever-growing public health problem (2).

Developed from the monocyte–macrophage hematopoietic lineage cell, osteoclasts adhere to the bone and secrete acid to dissolve the bone mineral. Then, they will undergo apoptosis after resorption (3, 4). Attenuated monocyte apoptosis has been demonstrated to be critical in osteoporosis (5). A higher percentage of monocytes may be a potential predictor of rheumatic diseases and osteoporosis (6, 7). Changes in gene or miRNA expression were observed in different samples, such as bone trabecula, serum, as well as peripheral blood monocytes (8–10). Moreover, a network analysis of gene modules related to bone mineral density demonstrated that particular expression of monocytes affects bone mass (11).

According to the surface antigens, human monocyte subsets can be divided into classical (CD14+CD16−), intermediate (CD14+CD16+), and non-classical (CD14−CD16+) monocytes, which have different abilities to secret cytokines (12). Classical monocytes play a vital role in the initial inflammatory response and cause chronic diseases. Intermediate and non-classical monocytes are grouped together as CD16+ monocytes in many studies. Non-classical monocyte functions are less clear in chronic disease, although their roles in atherosclerosis, ischemic reperfusion injury, rheumatoid arthritis and other disease have been reported (13). The composition of CD14+CD16+ monocytes was increased in multiple myeloma patients with osteolytic bone disease. Therefore, CD14+CD16+ monocyte might become a candidate marker for osteolytic bone destruction (14).

Monocytes serve as early progenitors of osteoclasts, which alter bone metabolism and bone mass. It is necessary to elucidate the role of monocytes in the progression of osteoporosis. Recent evidences have emerged that explorations of gene profile by bioinformatics analyses promote investigation of the molecular mechanisms for kinds of disease (15, 16). However, the relationship between osteoporosis and monocytes subsets gene variation is rarely reported. Therefore, we identified low-peak bone mass (LPBM) and CD16+ monocytes through cross-validation data sets Co-expressed genes in subpopulations. Based on the identified differentially expressed genes (DEGs), we screened out the hub genes and predicted their targeted miRNA. We constructed a CD16+ monocytes subsets miRNA-mRNA regulatory network in the context of LPBM, which might provide a new insight into pathophysiological mechanism and therapy for osteoporosis.

## Materials and Methods

### Microarray Data Source

Gene expression profiles were downloaded from the Gene Expression Omnibus (GEO, https://www.ncbi.nlm.nih.gov/geo/). The microarray profile dataset GSE7158, deposited by Lei et al., was conducted on circulating monocytes from 14 samples of extremely high-peak bone mass (HPBM) and 12 samples of extremely low-peak bone mass (LPBM). The dataset was based on the GPL570 [HG-U133_Plus_2] Affymetrix Human Genome U133 Plus 2.0 Array platform. Besides, the gene expression dataset GSE101489, provided by Cole S et al., was selected for containing CD16− classical monocytes samples and CD16+ non-classical monocytes samples. The dataset was based on the GPL10904 Illumina HumanHT-12 V4.0 expression beadchip platform. The miRNA expression profile GSE93883 included 12 patients with osteoporosis (six samples with fracture and six samples without fracture) and six non-osteoporotic patients. This dataset was based on GPL18058 Exiqon miRCURY LNA microRNA array, 7th generation [miRBase v18, condensed Probe_ID version] platform.

### Data Processing

The online analysis tool GEO2R (https://www.ncbi.nlm.nih.gov/geo/geo2r/) was used to detect the DEGs between HPBM and LPBM samples. Genes that met the criteria, the *P*-value <0.05, |log2 (Fold-Change)|≥ 1 were considered as DEGs. Venn diagram webtool (http://bioinformatics.psb.ugent.be/webtools/Venn/) was used to identify the related genes.

### Functional and Pathway Enrichment Analysis

Metascape online database (17) (http://metascape.org) was used for Gene Ontology (GO) annotation analysis of DEGs and Kyoto Encyclopedia of Genes and Genomes (KEGG) pathway enrichment analysis. GO annotation analysis includes biological process (BP), molecular function (MF), and cellular component (CC) (18). Using KEGG pathway analysis, a set of molecular interaction, reaction and relationship networks of DEGs were constructed (19). The gene expression information of all peak bone mass samples was uploaded to the GSEA software for further analysis (20, 21). The hallmark gene set database was selected as the reference gene set. The gene set was filtered with the minimum number of 15 genes and the maximum number of 500 genes by default. Normalized enrichment score (NES) >1, Nominal P-value <0.01, and FDR q-value <0.25 were set as the cut-off criteria.

### Protein−Protein Interaction Network Analysis and Hub Gene Identification

The Search Tool for the Retrieval of Interacting Genes (STRING) database (http://string-db.org/) was used to construct PPI network (22). To identify hub genes, the cytoHubba plugin was used in the Cytoscape software (version 3.7.2) based on the nodal degree (the number of genes connected to the target gene).

### Prediction of Potential miRNA

TargetScan, miRDB and miRWalk databases were used to predict target miRNAs (23–25). Only the intersection of the miRNA which expressed in all the three database and the differentially expressed miRNA (DEmiRNA) in GSE93883 was considered the target miRNA.

## Results

### Identification and Enrichment of Differentially Expressed Genes in Low-Peak Bone Mass Monocytes

**Figure 1:**
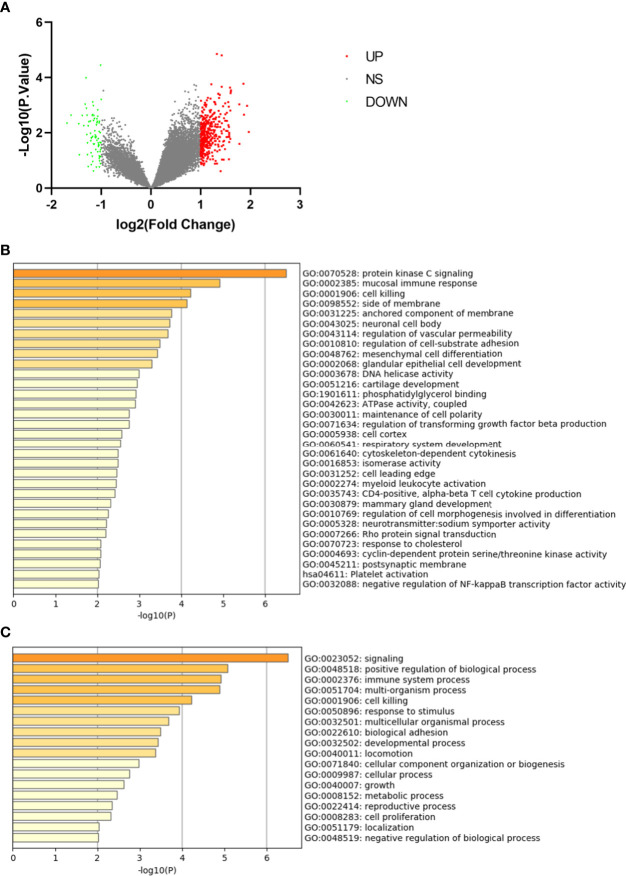
Differentially expressed genes in monocytes from low-peak bone mass. **(A)** Volcano plot for differentially expressing genes between low and high-peak bone mass samples. Red dots represent significantly up-regulated genes, green dots represent significantly down-regulated genes. **(B)** Heatmap of Gene Ontology (GO) and Kyoto Encyclopedia of Genes and Genomes (KEGG) enriched clusters colored by P-value. **(C)** The top-level Gene Ontology biological processes.

In the dataset GSE7158 contained 12 LPBM samples and 14 HPBM samples, a total of 368 DEGs were identified, including 308 up-regulated genes and 60 down-regulated genes (**Figure 1A**).

The GO and KEGG enrichment analyses of DEGs were performed using Metascape online tools. In the enriched functional category terms, 31 enriched GO terms and one KEGG pathway were identified (**Figure 1B**). KEGG pathway analysis showed that platelet activation was the most significant pathway. Enriched GO terms are presented in **Table 1**. The most affected GO biological processes included signaling, positive regulation of biological process and immune system process (**Figure 1C**). The network of GO and KEGG enriched terms were visualized by P-value (**Supplemental Figure 1A**) and clusters (**Supplemental Figure 1B**).

**Table 1:** Top 20 enriched terms for differentially expressed genes in monocytes of low peak bone mass (LPBM).

GO	Category	Description	Count	%	Log10(P)	Log10(q)
GO:0070528	GO Biological Processes	protein kinase C signaling	7	1.98	–6.5	–2.14
GO:0002385	GO Biological Processes	mucosal immune response	6	1.69	–4.92	–1.21
GO:0001906	GO Biological Processes	cell killing	11	3.11	–4.23	–0.82
GO:0098552	GO Cellular Components	side of membrane	22	6.21	–4.13	–0.77
GO:0031225	GO Cellular Components	anchored component of membrane	10	2.82	–3.76	–0.58
GO:0043025	GO Cellular Components	neuronal cell body	19	5.37	–3.72	–0.57
GO:0043114	GO Biological Processes	regulation of vascular permeability	5	1.41	–3.67	–0.54
GO:0010810	GO Biological Processes	regulation of cell-substrate adhesion	11	3.11	–3.48	–0.5
GO:0048762	GO Biological Processes	mesenchymal cell differentiation	11	3.11	–3.43	–0.5
GO:0002068	GO Biological Processes	glandular epithelial cell development	4	1.13	–3.29	–0.47
GO:0003678	GO Molecular Functions	DNA helicase activity	6	1.69	–2.99	–0.2
GO:0051216	GO Biological Processes	cartilage development	10	2.82	–2.95	–0.18
GO:1901611	GO Molecular Functions	phosphatidylglycerol binding	3	0.85	–2.92	–0.16
GO:0042623	GO Molecular Functions	ATPase activity, coupled	12	3.39	–2.9	-0.16
GO:0030011	GO Biological Processes	maintenance of cell polarity	3	0.85	–2.75	-0.04
GO:0071634	GO Biological Processes	regulation of transforming growth factor beta production	4	1.13	–2.75	–0.04
GO:0005938	GO Cellular Components	cell cortex	12	3.39	–2.58	0
GO:0060541	GO Biological Processes	respiratory system development	9	2.54	–2.55	0
GO:0061640	GO Biological Processes	cytoskeleton-dependent cytokinesis	6	1.69	–2.49	0
GO:0016853	GO Molecular Functions	isomerase activity	8	2.26	–2.49	0

“Count” is the number of genes in the 368 genes. “%” is the percentage of all of the 368 genes that are found in the given ontology term. “Log10(P)” is the p-value in log base 10. “Log10(q)” is the multi-test adjusted p-value in log base 10.

### GSEA of Low-Peak Bone Mass-Related Genes

**Figure 2:**
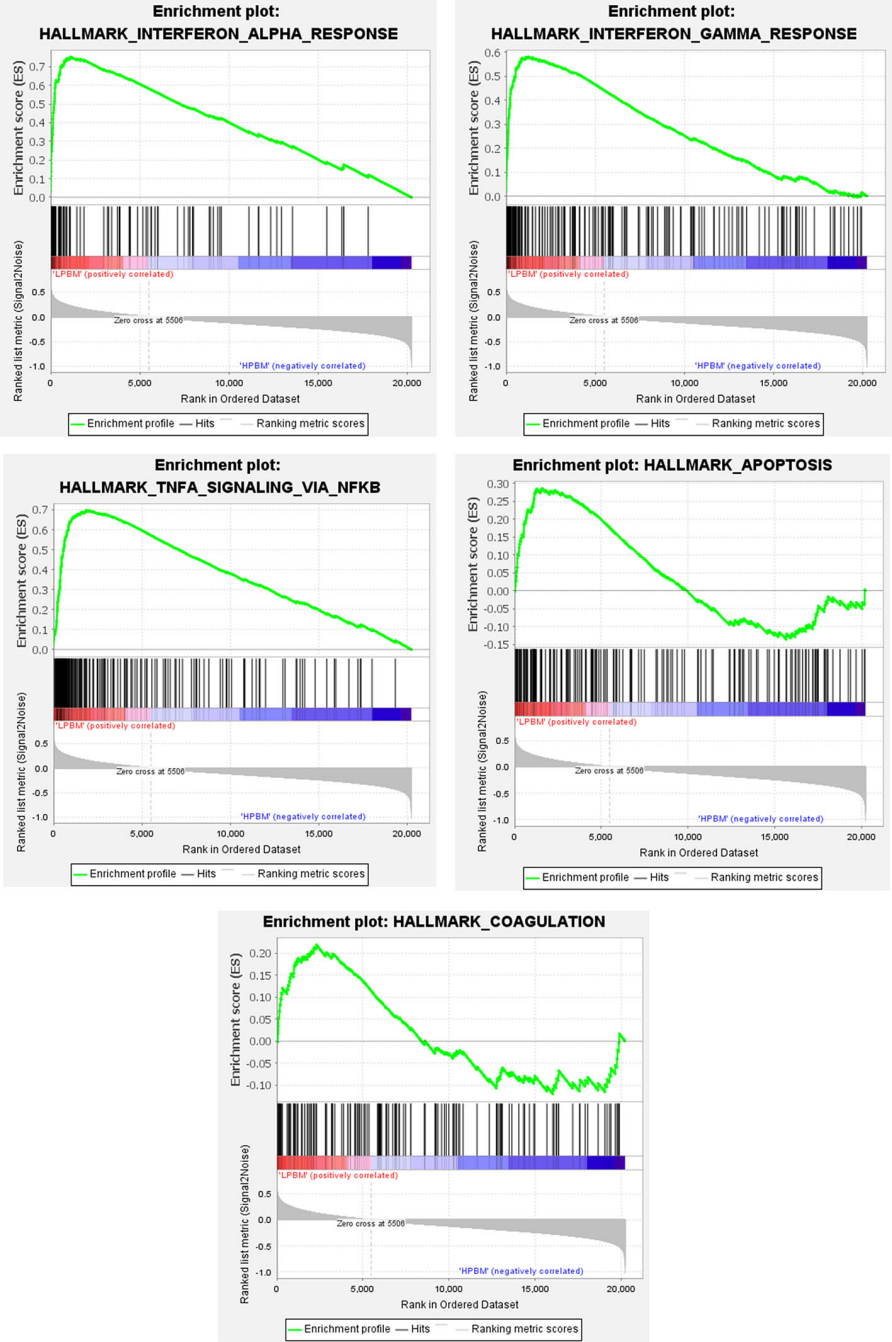
The whole gene expression value of low and high-peak bone mass samples was analyzed using GSEA software. Significant gene set criteria were set as NES >1, FDR <0.25 and P-value <0.01.

The Molecular Signatures Database was used to analyze genes in expression profile at a holistic level using GSEA software. Compared with HPBM samples a total of 12 gene sets were up-regulated in the LPBM samples, of which 10 gene sets were significantly enriched at FDR <0.25 and five gene sets were significantly enriched at nominal P value <0.01. As shown in **Figure 2**, the significant gene sets were enriched in interferon-*α*/*γ* response, TNF-α signaling *via* NF-*κ*B, apoptosis, and coagulation.

### Protein–Protein Interaction Network Analysis and Hub Gene Identification of Differentially Expressed Genes in Low-Peak Bone Mass Monocytes

**Figure 3:**
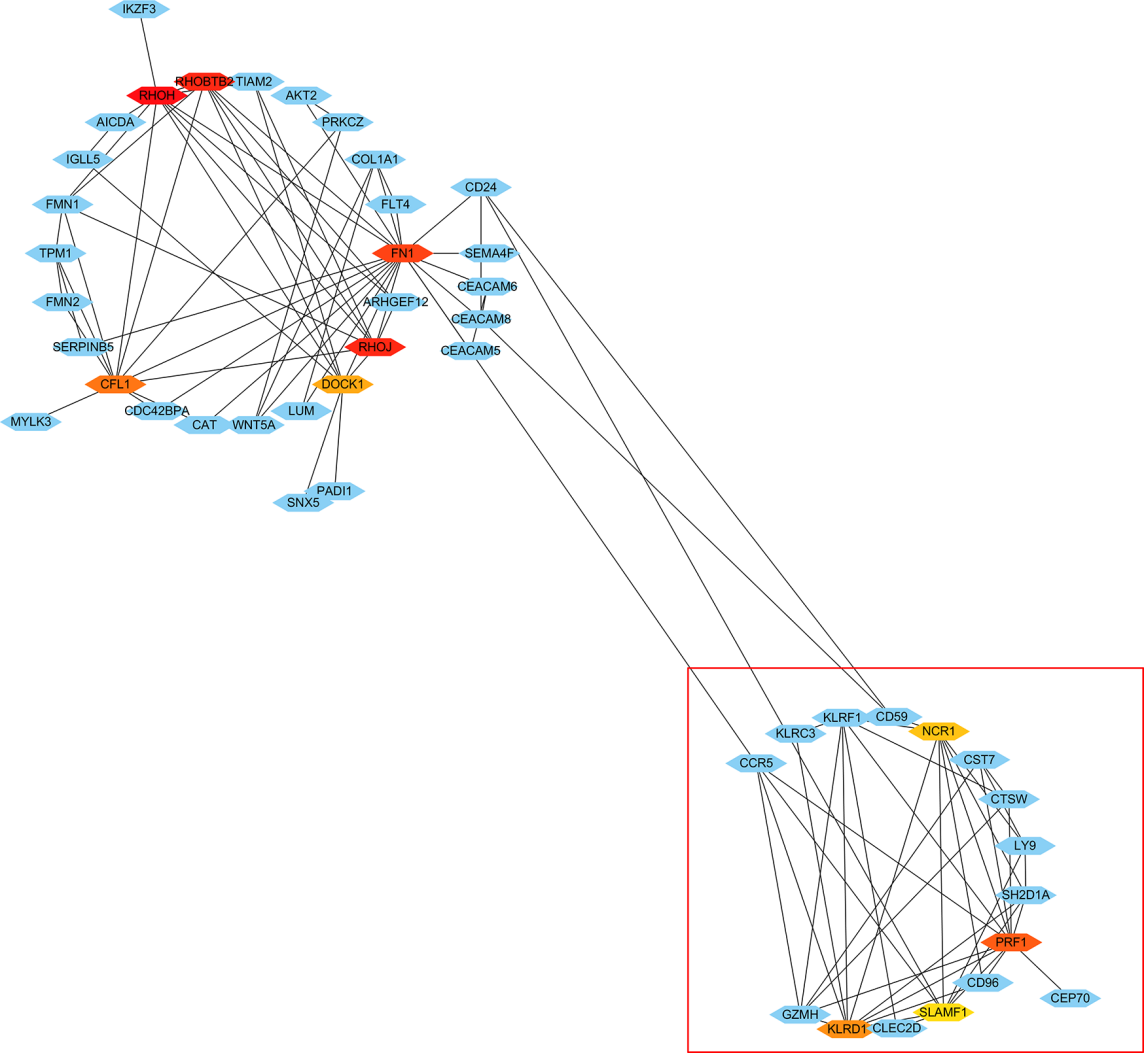
Cytoscape network clustering visualization of hub DEGs in monocytes from low-peak bone mass samples. The nodes represent genes and the edges represent links between genes. The red/orange/yellow color means the MMC scores is highest/moderate high/mild high.

Using the STRING tools, a total of 294 nodes and 298 edges were predicted in the PPI network with interaction ≥0.4 (**Supplemental Figure 2**). In order to identify hub genes in network, the cytoHubba plugin in Cytoscape software was used to cluster the network genes. The 10 hub genes with the highest MMC scores are listed in **Table 2**. As shown in **Figure 3**, the top 10 hub genes with their neighbors and expended genes were divided into two linkage modules.

**Table 2:** The hub genes for DEGs in monocytes of LPBM.

Rank	Gene Symbol	Gene Name	MMC Score
1	RHOH	Ras homolog family member H	104
2	RHOJ	Ras Homolog Family Member J	102
2	RHOBTB2	Rho Related BTB Domain Containing 2	102
4	FN1	Fibronectin 1	68
5	PRF1	Perforin 1	67
6	CFL1	Cofilin 1	58
7	KLRD1	Killer Cell Lectin Like Receptor D1	56
8	DOCK1	Dedicator of Cytokinesis 1	51
9	NCR1	Natural Cytotoxicity Triggering Receptor 1	43
10	SLAMF1	Signaling Lymphocytic Activation Molecule Family Member 1	38

### Identification and Enrichment of Differentially Expressed Genes in CD16+ Monocytes

**Figure 4:**
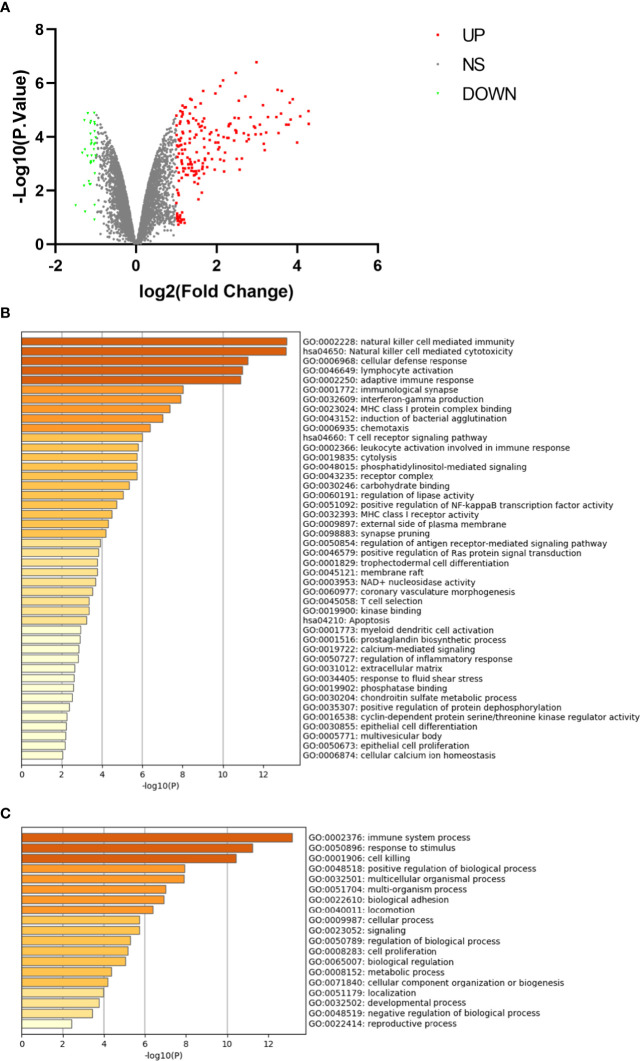
Differentially expressed genes in CD16+ monocyte samples. **(A)** Volcano plot for differentially expressing genes between CD16+ and CD16− monocyte samples. Red dots represent significantly upregulated genes, green dots represent significantly downregulated genes. **(B)** Heatmap of Gene Ontology (GO) and Kyoto Encyclopedia of Genes and Genomes (KEGG) enriched clusters colored by P-value. **(C)** The top-level Gene Ontology biological processes.

The microarray expression dataset GSE101489 was download. The analysis was performed to acquire DEGs between CD16+ monocytes and CD16- monocytes (**Figure 4A**). A total of 201 DEGs were identified including 169 up-regulated genes and 32 down-regulated genes. Enriched GO terms and KEGG pathways were identified using Metascape online tools. Natural killer cell mediated cytotoxicity, T cell receptor signaling pathway and apoptosis were significantly enriched in the KEGG pathway (**Figure 4B**). The enriched GO terms included immune system process, response to stimulus and cell killing (**Figure 4C**). The enriched terms are presented in **Table 3**.

**Table 3:** Top 20 enriched terms for differentially expressed genes in CD16+ monocytes.

GO	Category	Description	Count	%	Log10(P)	Log10(q)
GO:0002228	GO Biological Processes	natural killer cell mediated immunity	12	6.63	–13.17	–9.08
hsa04650	KEGG Pathway	Natural killer cell mediated cytotoxicity	15	8.29	–13.14	–9.08
GO:0006968	GO Biological Processes	cellular defense response	10	5.52	–11.23	–7.35
GO:0046649	GO Biological Processes	lymphocyte activation	27	14.92	–10.95	–7.22
GO:0002250	GO Biological Processes	adaptive immune response	26	14.36	–10.88	–7.22
GO:0001772	GO Cellular Components	immunological synapse	7	3.87	–8.02	–4.87
GO:0032609	GO Biological Processes	interferon-gamma production	10	5.52	–7.89	–4.79
GO:0023024	GO Molecular Functions	MHC class I protein complex binding	4	2.21	–7.36	–4.4
GO:0043152	GO Biological Processes	induction of bacterial agglutination	4	2.21	–6.99	–4.11
GO:0006935	GO Biological Processes	chemotaxis	19	10.5	–6.39	–3.64
hsa04660	KEGG Pathway	T cell receptor signaling pathway	8	4.42	–5.99	–3.31
GO:0002366	GO Biological Processes	leukocyte activation involved in immune response	19	10.5	–5.8	–3.14
GO:0019835	GO Biological Processes	cytolysis	5	2.76	–5.74	–3.1
GO:0048015	GO Biological Processes	phosphatidylinositol-mediated signaling	10	5.52	–5.74	–3.1
GO:0043235	GO Cellular Components	receptor complex	16	8.84	–5.72	–3.09
GO:0030246	GO Molecular Functions	carbohydrate binding	11	6.08	–5.33	–2.78
GO:0060191	GO Biological Processes	regulation of lipase activity	7	3.87	–5.03	–2.54
GO:0051092	GO Biological Processes	positive regulation of NF-kappaB transcription factor activity	8	4.42	–4.71	–2.26
GO:0032393	GO Molecular Functions	MHC class I receptor activity	3	1.66	–4.48	–2.11
GO:0009897	GO Cellular Components	external side of plasma membrane	12	6.63	–4.3	–1.94

“Count” is the number of genes in 201 genes. “%” is the percentage of all of the 201 genes that are found in the given ontology term. “Log10(P)” is the p-value in log base 10. “Log10(q)” is the multi-test adjusted p-value in log base 10.

### Protein–Protein Interaction Network Analysis of Differentially Expressed Genes in CD16+ Monocytes

**Figure 5:**
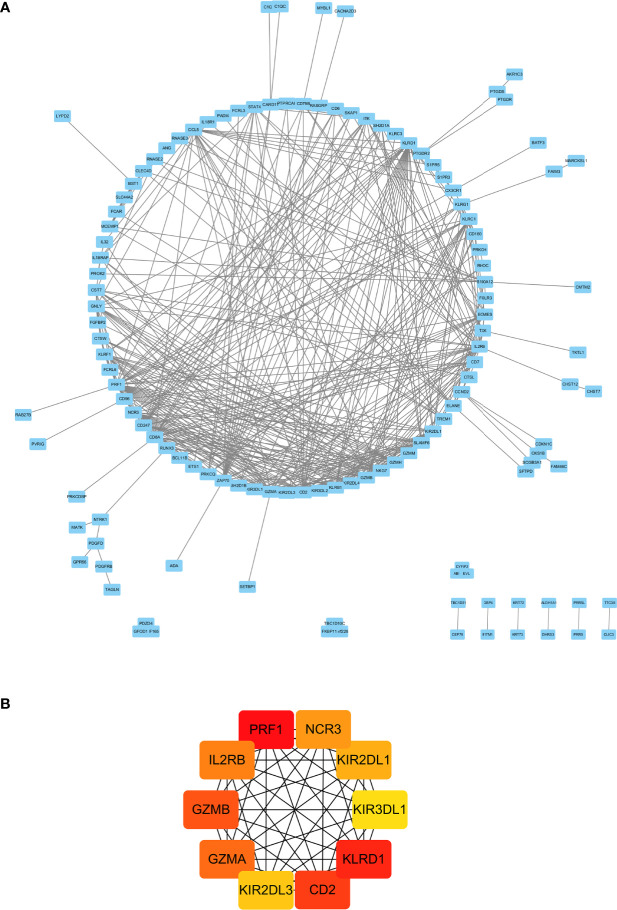
PPI network analysis of DEGs in CD16+ monocytes. **(A)** PPI network of differentially expression genes in CD16+ monocytes. **(B)** Hub genes in the PPI network.

A PPI network with an interaction score >0.4 was obtained based on the STRING online database (**Figure 5A**). The hub genes were clustered using the cytoHubba plugin in Cytoscape software. As shown in **Figure 5B**, the top 10 nodes ranked by MCC were clustered. Moreover, all of them were the up-regulated genes in CD16+ monocytes.

### Identification and Cluster Analysis of Overlapping Genes

**Figure 6:**
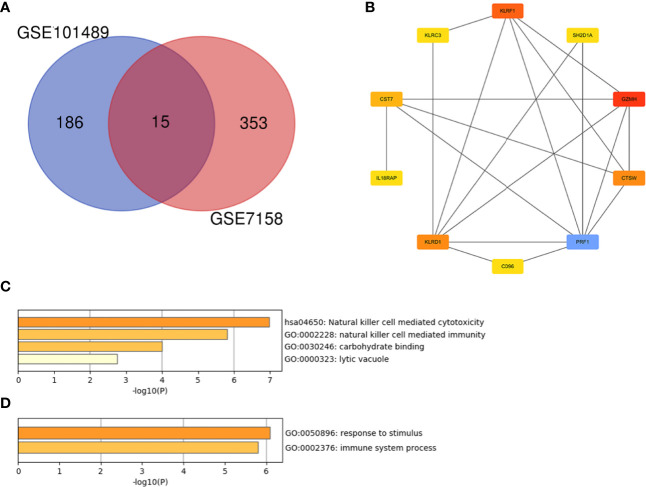
**(A)** Venn diagram of differentially expressed genes between the low-peak bone mass in GSE7158 and CD16+ monocytes subset in GSE101489. **(B)** Analysis of hub genes using cytoHubba for the 15 overlapping genes. **(C)** Heatmap of Gene Ontology (GO) and Kyoto Encyclopedia of Genes and Genomes (KEGG) enriched clusters colored by P-value. **(D)** The top-level Gene Ontology biological processes.

As shown in **Figure 6A**, there were 15 genes overlapping between the 368 DEGs in LPBM and 201 DEGs in CD16+ monocytes. Moreover, all the 15 genes were the up-regulated DEGs in both datasets. Then, cluster analysis was performed to visualize the interactional relationship between the LPBM and CD16+ monocytes subset. Network analysis and gene clustering were carried out using Cytoscape software. In the **Figure 6B**, ten key PPI modules were identified from the 15 overlapping genes. Moreover, nine of them (KLRF1, GZMH, CTSW, KLRD1, CST7, KLRC3, SH2D1A, CD96, PRF1) existed in a hub gene module of LPBM DEGs (a red box in **Figure 3**). It reveals that the nine genes in CD16+ monocytes play an important role in the process of bone mass loss.

Further functional enrichment analysis for the nine hub genes were performed using Metascape online tool. The natural killer cell mediated cytotoxicity was significantly enriched in the KEGG pathway (**Figure 6C**). The enriched terms are presented in **Table 4**. The GO terms included carbohydrate binding in the MF category, lytic vacuole in the CC category and natural killer cell mediated immunity in the MF category. The top-level GO biological processes were present in **Figure 6D**, including response to stimulus and immune system process.

**Table 4:** The enriched terms for 15 overlapping genes.

GO	Category	Description	Count	%	Log10(P)	Log10(q)
hsa04650	KEGG Pathway	Natural killer cell mediated cytotoxicity	4	44.44	–6.98	–2.62
GO:0002228	GO Biological Processes	natural killer cell mediated immunity	3	33.33	–5.8	–1.92
GO:0030246	GO Molecular Functions	carbohydrate binding	3	33.33	–4	–0.34
GO:0000323	GO Cellular Components	lytic vacuole	3	33.33	–2.76	0

“Count” is the number of genes in the nine genes. “%” is the percentage of all of the 9 genes that are found in the given ontology term. “Log10(P)” is the p-value in log base 10. “Log10(q)” is the multi-test adjusted p-value in log base 10.

### Prediction and Confirmation of Target miRNA for Hub Genes

Based on the miRDB, TargetScan and miRWalk online databases, 467, 4,008, and 5,540 target miRNAs were predicted for the nine hub genes. After intersection, 183 miRNAs were selected, which were all expressed in three databases.

**Figure 7:**
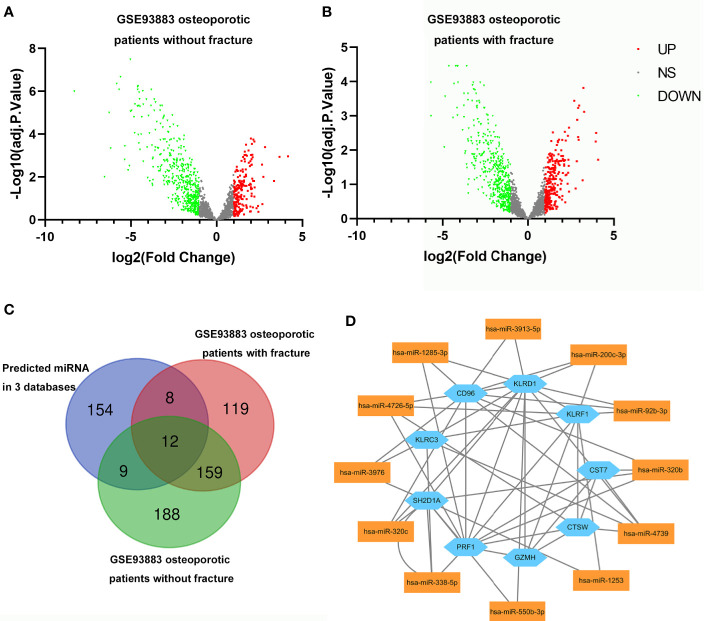
Volcano plots for differentially expressing miRNAs (DEmiRNAs) in osteoporotic patients without **(A)**/with fracture **(B)**. Red dots represent significantly upregulated miRNAs, green dots represent significantly downregulated miRNAs. **(C)** Venn diagram showing the numbers of predicted miRNAs, DEmiRNAs in osteoporotic patients with fracture and those without fracture from GSE93883 dataset. **(D)** The relationship between nine hub genes and 12 selected miRNAs (miRNA-mRNA regulatory network).

The DEmiRNAs in primary osteoporotic patients without or with vertebral fractures were obtained by analyzing the GSE 93883 dataset (**Figures 7A, B**). After intersection with the 183 predicted miRNAs, 12 miRNAs were screen out (**Figure 7C**). The miRNA–mRNA regulatory network of monocytes in patients with osteoporosis was established based on the predicted miRNA–RNA pairs (**Figure 7D**).

## Discussion

Osteoporosis is a common disease characterized by reduced bone mass, deteriorated bone tissue and increased susceptibility to fracture. Monocytes are the largest type of leukocytes, which can differentiate into myeloid lineage dendritic cells and macrophages. Cytokines and growth factors secreted by monocytes can affect bone (26, 27). In addition, osteoclast is a kind of bone-resorbing macrophage polykaryon, which is differentiated from monocyte/macrophage precursor (28, 29). Therefore, monocytes have been studied with the osteoporotic pathophysiology for several years (30, 31). Distinct population of monocytes display considerable heterogeneity based on their phenotype and function (32). Hence, we hypothesized that the phenotypes of LPBM were influenced by the aberrant expansion of monocyte subsets. However, the relationship between the gene expression profile of monocytes subsets and the pathogenesis of osteoporosis is rarely reported. To support this hypothesis, we identified 368 DEGs in monocytes of peak bone mass (LPBM *vs.* HPBM) patients, 201 DEGs in monocyte subsets (CD16+ *vs.* CD16−) through integrative analysis of GEO data. In order to validate the biologic relevance of these modules, the intersection of DEGs in the two series were performed and 15 overlapping DEGs were screened out. Moreover, the 15 genes were up-regulated in both datasets. PPI analysis and hub gene identification revealed that nine key genes in monocytes (KLRF1, GZMH, CTSW, KLRD1, CST7, KLRC3, SH2D1A, CD96, PRF1) might play a vital role in osteoporosis. Based on the prediction of hub genes and cross-validation with miRNA expression profile, 12 miRNAs were picked out and a potential miRNA–mRNA network was constructed.

The subjects with high or low peak bone mass in GSE7158 were recruited from a healthy premenopausal female population. At the age from 20 to 45 years, women’s bone mineral density (BMD) is in a relative balance because the peak bone mass is reached (33). The expression of relevant genes which reflects bone metabolism is much less influenced by hormones and nutrition. Therefore, it would be appropriate opportunity to explore the genetic factors responsible for BMD variation at peak bone mass (34). Hundreds of genes have been confirmed to be involved as osteoporosis is a polygenetic disease. Pathways related to bone metabolism and immune system were reported to be involved in the biological processes, including extracellular matrix metabolism, the cytokine and cytokine receptor network, as well as Wnt signaling and so on (35). Previous study demonstrated that interferon could induce vital intermediate expression in osteoblasts in response to the stimulation for proliferation/differentiation (36). Blocking the TNF-α pathway could prevent bone loss in murine model for hemophilic arthropathy (37). Consistent with our data, GSEA analysis results showed that interferon-*α/γ* response, TNF-α signaling *via* NF-*κ*B, apoptosis and coagulation were the significantly enriched pathways. Additionally, our enrichment analysis demonstrated that DEGs between HPBM and LPBM were mainly enriched in signaling, positive regulation of biological process and immune system process. Therefore, our data-mining results confirmed that the immune system played a critical role in the etiology of osteoporosis. It was consistent with previous report that cytokines and immune factors produced by immune cells could regulate bone resorption and formation (38). Such as T cells could regulate bone homeostasis by secreting pro-osteoclastogenic cytokines and anti-osteoclastogenic cytokines (39–41). Moreover, bone homeostasis could be regulated by immune cells of the bone marrow (42).

Inflammation and the innate defense in mammals can be linked to adaptive immune responses through monocytes, which means that monocytes is a kind of accessory cell (43). Monocyte subsets are identified based on their surface antigens with unique transcriptional and functional characteristics (44). Although non-classical monocytes are widely regarded as protective cells, their role in chronic disease remains unclear. Previous study showed that aberrant expansion of (CD14+CD16+) monocyte subsets contributed to the enhanced apoptotic phenotype in the pathogenesis of IgA nephropathy (45). In the disease with inflammation-driven bone loss, the composition of CD14+CD16+ vs. CD14+CD16− monocytes altered, which led to an increase in osteoclast formation (46). Since CD16 is the main surface marker expressed by monocytes, these cell types deserve further analysis. However, monocyte subset gene identification for osteoporosis is rarely reported. Therefore, our research first time elucidated gene profile identification of monocyte subsets in different bone mass population by cross-validation of data. The functional analysis of DEGs on CD16+ monocytes in our study demonstrated that the most significant pathways were natural killer cell mediated cytotoxicity, T cell receptor signaling pathway and apoptosis. The enriched GO terms included immune system process, response to stimulus and cell killing. These enriched terms are similar to the enrichment analysis for osteoporosis above (35).

The enrichment analysis of nine hub genes in the 15 overlapping DEGs showed that the top one enriched KEGG pathway was natural killer cell mediated cytotoxicity. According to the GeneCards database (https://www.genecards.org/), all the nine genes play a vital role in immunity. Killer Cell Lectin Like Receptor D1(KLRD1), Killer Cell Lectin Like Receptor F1 (KLRF1), Killer Cell Lectin Like Receptor C3 (KLRC3) and Granzyme H (GZMH) are expressed primarily in natural killer (NK) cells and participate in cytokine release. NK cells mediate cytotoxic activity and secrete cytokines in response to immune stimulation. Cathepsin W (CTSW) may play a role in the regulation of T-cell cytolytic activity. SH2 Domain Containing 1A (SH2D1A) can modify signal transduction pathways by binding to the surface molecules on activated T, B, and NK cells. CD96 participates in the adhesion interaction between activated T cells and NK cells in the immune response. Perforin 1 (PRF1) participates in killing other cells that cannot be recognized as families. Cystatin F (CST7) may inhibit a unique target in the hematopoietic system and regulate immune process. In addition, the nine genes all existed in one modules of hub gene for DEGs of LPBM. Therefore, we speculated that the up-regulated nine key genes in CD16+ monocytes might play a vital role in immune response during bone loss process. The datasets for peak bone mass and miRNAs profiles in osteoporotic patients were all from Asian population. Therefore, the key genes identified in this study will be further investigated with human samples in follow-up study.

In summary, we screened out 15 DEGs from two datasets and identified nine hub genes by constructing a PPI network. Moreover, 12 target miRNAs were predicted with three databases and confirmed using miRNA expression profiling data. Then, a miRNA–mRNA network was constructed. Our study provided a reliable comprehensive analysis on the DEGs profile in monocyte subsets for bone loss. Further studies are required to explore the mechanism of these potential genes associated with osteoporosis in CD16+ monocytes.

## Data Availability Statement

The original contributions presented in the study are included in the article/**Supplementary Material**; further inquiries can be directed to the corresponding authors.

## Author Contributions

BH analyzed part of the data and wrote the manuscript. XK, LL, and FD collected the data and analyzed part of the data. QZ contributed to the discussion and reviewed the manuscript. RS involved in the overall study, designed the analysis plan, performed the statistical analyses guidance and revised the manuscript. All authors contributed to the article and approved the submitted version.

## Conflict of Interest

The authors declare that the research was conducted in the absence of any commercial or financial relationships that could be construed as a potential conflict of interest.
