# Supplementary figures and images for: Integrative Analyses of Genes Associated With Osteoporosis in CD16+ Monocyte
Source: Front Endocrinol (Lausanne). 2021 Jan 21;11:581878. doi: 10.3389/fendo.2020.581878 (PMC7859337; doi:10.3389/fendo.2020.581878)

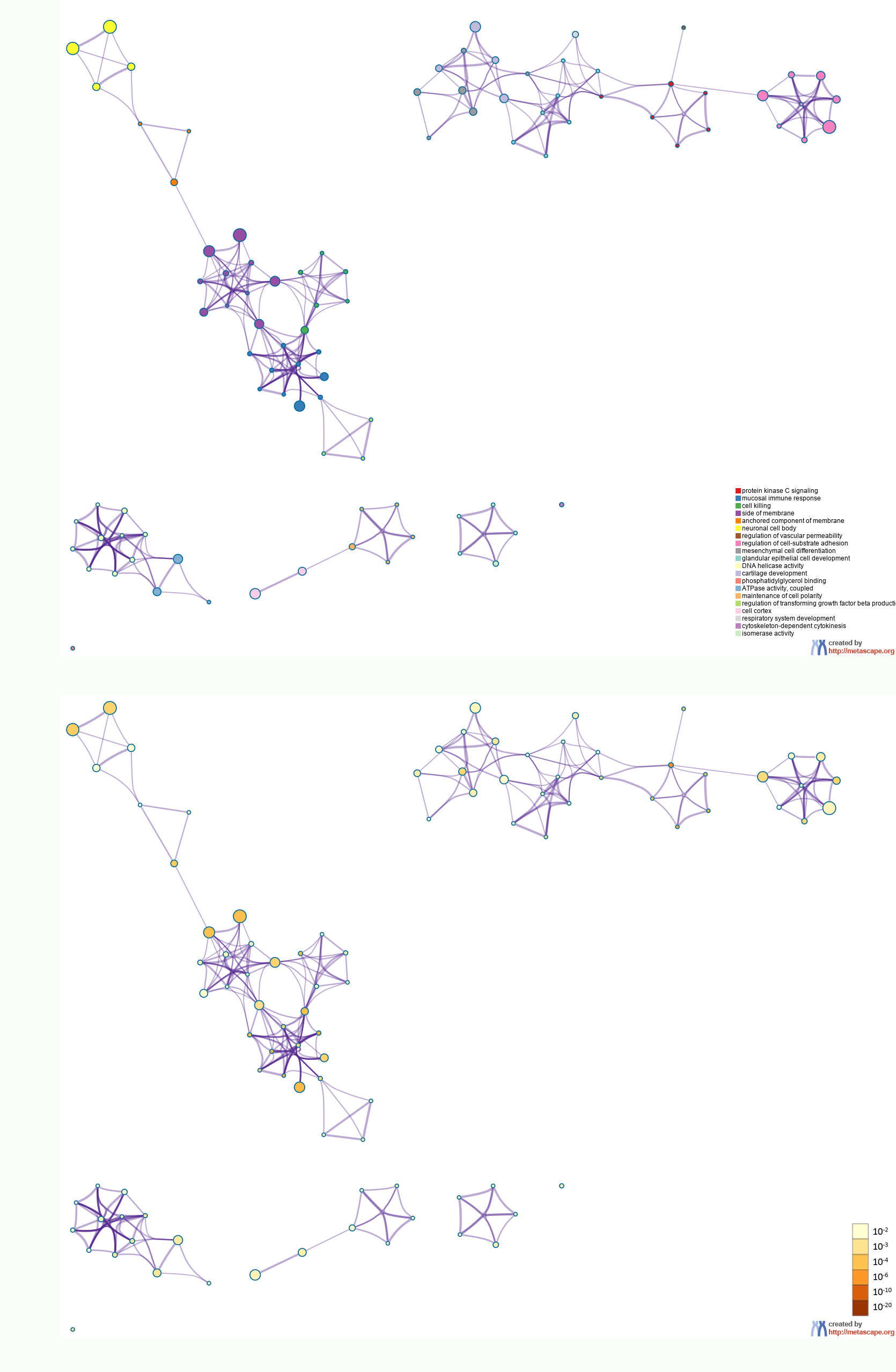

Supplement: Supplementary file 1 [file Image_1.tif]

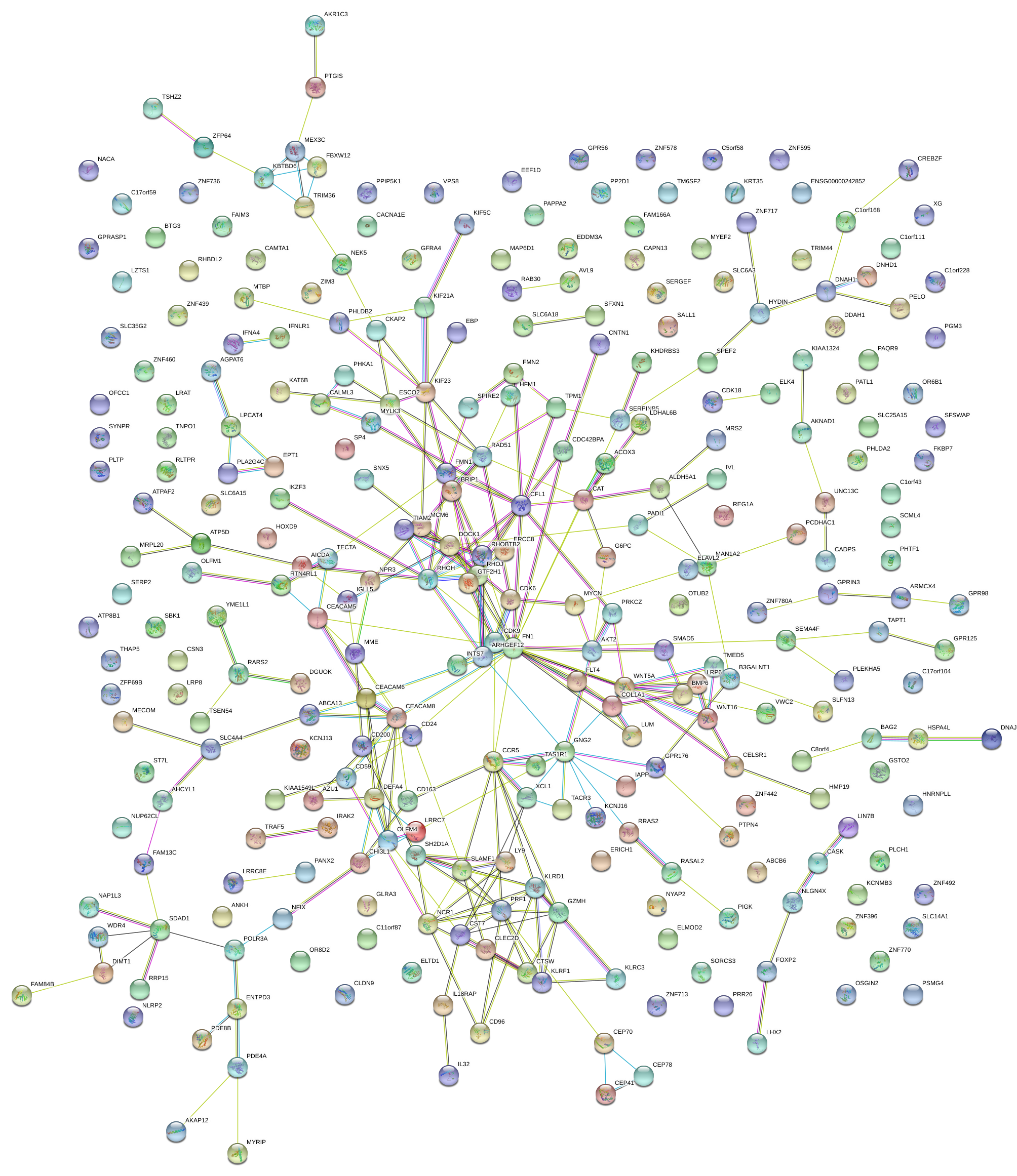

Supplement: Supplementary file 2 [file Image_2.png]
